# Prevalence of drug resistant Enterobacteriaceae in a Nepalese tertiary care hospital

**DOI:** 10.1371/journal.pgph.0000858

**Published:** 2024-01-19

**Authors:** Anita Bhandari, Saroj Khatiwada, Aashish Sharma, Subhas Chandra Aryal, Raju Shrestha, Nabin Kishor Bimali, Binod Lekhak, Narayan Dutt Pant

**Affiliations:** 1 Department of Microbiology, GoldenGate International College, Tribhuvan University, Kathmandu, Nepal; 2 Department of Biochemistry, Modern Technical College, Lalitpur, Nepal; 3 Department of Microbiology, National College, Tribhuvan University, Kathmandu, Nepal; 4 Central Department of Microbiology, Tribhuvan University, Kathmandu, Nepal; 5 Grande Hospital, Kathmandu, Nepal; University of New Brunswick Saint John, CANADA

## Abstract

Antimicrobial resistance in Enterobacteriaceae is an emerging global public health problem. Numerous studies have reported community-acquired AmpC beta-lactamase and extended spectrum beta-lactamase (ESBL) producing Enterobacteriaceae in Nepal. However, there are limited data on community-acquired Metallo-beta-lactamase (MBL) producing Enterobacteriaceae. A hospital-based descriptive cross-sectional study was conducted using 294 Enterobacteriaceae isolates from a total of 2,345 different clinical specimens collected from patients attending a tertiary care hospital in Nepal. Bacteria were isolated using standard microbiological growth media and identified using biochemical tests. For antimicrobial susceptibility testing, Kirby-Bauer disc diffusion technique was used. AmpC, ESBL, and MBL productions were detected by using combined disc method. AmpC, ESBL, and MBL productions were detected in 19.4%, 29.6%, and 8.5% of total Enterobacteriaceae isolates respectively. Higher rates of beta-lactamases production were seen among the isolates from in-patients in comparison with those from out-patients. However, 11.6%, 25%, and 3.7% of the total isolates from out-patients were AmpC, ESBL, and MBL producers respectively. The co-production of the beta-lactamases was also detected, with two *Klebsiella pneumoniae* isolates producing all three beta-lactamases. One MBL producing *Proteus vulgaris* isolate that was pan-resistant with no remaining treatment options was also isolated. Prevalence of drug resistant Enterobacteriaceae in our study was very high. Detection of AmpC, ESBL, and MBL positive isolates from out-patients, who did not have recent history of hospital visit, indicated the community dissemination of the drug resistant bacteria. This is a matter of great concern and an immediate attention to formulate strategies to prevent further development and spread of antibiotic resistance is required.

## Introduction

Antimicrobial resistance is a major global public health problem [1]. Beta-lactam antibiotic resistance in Enterobacteriaceae is increasingly being reported from both critically ill hospitalized patients and outpatients [2]. In 2015, the global prevalence of third generation cephalosporin resistance and carbapenem resistance among *Escherichia coli* were 64.5% and 5.8% and that for *Klebsiella pneumoniae* were 66.9% and 23.4% respectively [3]. Similarly by 2030, the prevalence of third generation cephalosporin resistance and carbapenem resistance among *E*. *coli* were estimated to reach 77% and 11.8% and those for *K*. *pneumoniae* to reach 58.2% and 52.8% respectively [3]. The main reason for β-lactam resistance among Enterobacteriaceae is the production of beta-lactamases, such as AmpC, ESBLs, and MBLs [4].

AmpC beta-lactamases are encoded on both chromosomes and plasmids and confer resistance to cephalothin, cefazolin, cefoxitin, most penicillins, monobactams, and β-lactamase inhibitor-β-lactam combinations [5–7]. The mechanisms of AmpC β-lactam resistance are inducible chromosomal resistance, non-inducible chromosomal resistance and plasmid-mediated resistance [8]. The high-level expression of inducible AmpC enzymes can cause resistance to broad-spectrum cephalosporins such as cefotaxime, ceftazidime, and ceftriaxone [5]. Inducible AmpC is a major problem in infections caused by members of Enterobacteriaceae, such as *Klebsiella aerogenes* and *Enterobacter cloacae* [5]. An isolate with inducible AmpC initially susceptible may develop resistance to broad-spectrum cephalosporins after exposure [5]. AmpC coding genes can easily transmit to other bacteria, such as *E*. *coli* and *K*. *pneumoniae*, through plasmids [7, 9]. AmpC producing bacteria are susceptible to fourth-generation cephalosporins, and carbapenems [6].

ESBL producing bacteria are resistant to penicillins, monobactams, and most of the cephalosporins including ceftazidime, cefotaxime, and ceftriaxone [10]. These plasmid-mediated resistance phenotypes can also be easily transmitted among different bacterial species and can confer resistance to other antibiotic classes including fluoroquinolones and aminoglycosides [9, 10]. Carbapenems are used for the treatment of AmpC and ESBL positive bacterial infections [11]. However, there are reports of the increased prevalence of MBL mediated carbapenem resistance globally, mainly in clinical settings [12]. There are very limited therapeutic options, such as tigecycline and colistin, for the treatment of infections caused by MBL producing bacteria [11]. However, for the bacteria such as MBL positive *Proteus* spp. which are intrinsically resistant to tigecycline and colistin, no treatment option is available [13].

In this study, we conducted a survey of Enterobacteriaceae among hospitalized patients and outpatients attending a tertiary care hospital in Nepal and determined phenotypic resistance level for commonly used extended spectrum beta lactams. This snapshot of resistance levels will inform (declining) treatment options for this community.

## Materials and methods

### Ethics statement

Ethical approval was taken from Nepal Health Research Council (reg. no. 447/2017).

### Study design

A hospital-based descriptive cross-sectional study was conducted in 2018, using 294 Enterobacteriaceae isolates isolated from 2,345 different clinical specimens (1013 from inpatients and 1332 from outpatients) from patients attending a tertiary care hospital in Nepal. For inpatients, the patients who showed the symptoms of infections for which they were not initially hospitalized at least 48hrs after hospital admission were included in this study. Additionally, patients known to be on antibiotic therapy in last one week before sample collection were excluded from the study. Bacterial colonies grown on standard microbiological culture media were identified with the help of biochemical tests as per Bergey’s manual [14, 15]. Colony characteristics, Gram staining, and biochemical tests—catalase, oxidase, sulfur indole motility test, methyl red test, voges-proskauer test, citrate utilization test, triple sugar iron agar test, urease test, and oxidative fermentative test were used to identify a bacterium to species level following Cheesbrough’s district laboratory practice in tropical countries part 2 [14]. For antimicrobial susceptibility testing, the Kirby-Bauer disk diffusion technique following Clinical and Laboratory Standards Institute (for all antibiotics used except tigecycline) and European Committee on Antimicrobial Susceptibility Testing guidelines was used (for tigecycline) [16, 17]. In short, a bacterial lawn culture was prepared on Mueller-Hinton agar plate from a broth culture with turbidity equivalent to 0.5 McFarland turbidity standard. Then antibiotic discs were put over the lawn culture by maintaining 24mm distance between center to center of two discs and the plate was incubated for 24hrs at 37°C. The antibiotic discs used were amoxicillin/clavulanic acid (20/10μg), piperacillin/tazobactam (100/10μg), cefoxitin (30μg), ceftazidime (30μg), cefotaxime (30μg), meropenem (10μg), imipenem (10μg), co-trimoxazole (23.75/1.25μg), nitrofurantoin (300μg), norfloxacin (10μg), levofloxacin (5μg), ciprofloxacin (5μg), gentamicin (30μg), amikacin (30μg), chloramphenicol (30μg), and tigecycline (15μg). The bacteria were reported as sensitive or resistant to the antibiotics on the basis of size of the inhibitory zone around the antibiotic discs. *E*. *coli* ATCC 25922 was used as control strain for susceptibility testing.

### Phenotypic detection of AmpC, ESBL, and MBL production

Isolates showing resistance to cefoxitin were screened as possible AmpC producers, which were later confirmed by combined disc assay using cefoxitin (30μg) and cefoxitin/ phenylboronic acid (30/20μg) [18]. A ≥5mm increase in the inhibition zone diameter of cefoxitin/phenylboronic acid compared with that of cefoxitin confirmed AmpC production. Similarly, ceftazidime resistant isolates screened as possible ESBL producers were confirmed using ceftazidime (30μg) and ceftazidime/clavulanic acid (30/10μg) [16]. A ≥5mm increase in the inhibition zone diameter of ceftazidime/clavulanic acid compared with that of ceftazidime confirmed ESBL production. Imipenem resistant isolates were confirmed for MBL production by using imipenem (10μg) and imipenem/ethylenediaminetetraacetate (10/750 μg) discs [19]. A ≥5mm increase in the inhibition zone diameter of imipenem/ethylenediaminetetraacetate compared with that of imipenem confirmed MBL production. *Enterobacter cloacae* ATCC BAA-1143, *Klebsiella pneumoniae* ATCC 700603, and *Klebsiella pneumoniae* ATCC BAA-2146 were used as positive quality control for AmpC, ESBL, and MBL production respectively and *E*. *coli* ATCC 25922 was used as negative quality control for all three beta-lactamases production assays.

### Statistical analysis

Data were analyzed using the statistical package for social science (SPSS) version 21. Chi-square test was used and p-value < 0.05 was considered significant.

## Results

Out of 2345 clinical samples, 483 (20.6%) were culture positive. Among culture positive samples, members of Enterobacteriaceae were isolated from 294 (60.9%) samples. Out of 294 Enterobacteriaceae isolates, 63.3% were isolated from urine, followed by sputum (20.7%) and blood (6.1%). Similarly, the most predominant isolates were *E*. *coli* (59.2%) followed by *K*. *pneumoniae* (30.6%) and *K*. *oxytoca* (4.1%).

Among a total of 294 Enterobacteriaceae isolates, 57 (19.4%) were AmpC positive, 87(29.6%) were ESBL positive, and 25 (8.5%) were MBL positive. The highest numbers of AmpC, ESBL, and MBL producing Enterobacteriaceae were isolated from urine followed by sputum. Among 186 Enterobacteriaceae isolated from urine, 12.4%, 33.9%, and 6.5% were AmpC, ESBL, and MBL positive respectively, while among 61 isolates from sputum, 32.8%, 26.2%, and 16.4% were AmpC, ESBL, and MBL positive respectively.

Among total Enterobacteriaceae isolated, 184 (62.6%) were multidrug-resistant. The highest numbers of *K*. *pneumoniae* isolates followed by *E*. *coli* were AmpC and MBL positive, while the highest numbers of *E*. *coli* isolates followed by *K*. *pneumoniae* were ESBL positive and multidrug-resistant. Among 174 *E*. *coli* isolates, 12.07%, 31.03%, and 1.7% respectively were AmpC, ESBL, MBL positive and 59.2% were multidrug-resistant. Similarly, among 90 *K*. *pneumoniae* isolates, 35.6%, 28.9%, and 23.3% respectively were AmpC, ESBL, MBL positive and 73.3% were multidrug-resistant (Table 1).

**Table 1:** AmpC, ESBL, MBL productions and multidrug resistance among different isolates.

Organisms	Total	AmpC (%)	ESBL (%)	MBL (%)	MDR (%)
*E*. *coli*	174	21 (12.07)	54 (31.03)	3 (1.7)	103 (59.2)
*K*. *pneumoniae*	90	32 (35.6)	26 (28.9)	21 (23.3)	66 (73.3)
*K*. *oxytoca*	12	2 (16.7)	3 (25)	-	7 (58.3)
*C*. *freundii*	5	1 (20)	1 (20)	-	2 (40)
*C*.*koseri*	2	-	-	-	-
*P*. *mirabillis*	4	1 (25)	2 (50)	-	3 (75)
*P*. *vulgaris*	3	-	1 (33.3)	1 (33.3)	3 (100)
*S*. Typhi	4	-	-	-	-
**Total**	**294**	**57(19.4)**	**87 (29.6)**	**25 (8.5)**	**184 (62.6)**

### Distribution of AmpC, ESBL, and MBL producing Enterobacteriaceae based on patient types

Higher numbers of bacteria isolated from inpatients were AmpC, 38(29.2%) vs 19 (11.6%), ESBL, 46 (35.4%) vs 41 (25%), and MBL, 19 (14.6%) vs 6 (3.7%) producers in comparison with those isolated from outpatients (p <0.05).

### Distribution of beta-lactamase co-producers among Enterobacteriaceae isolates

Beta-lactamase co-production was only seen in *E*. *coli* and *K*. *pneumoniae*. Among a total of 294 Enterobacteriaceae isolates, 2 (0.7%) isolates produced all three beta-lactamases, 6 (2.04%) isolates produced ESBL and MBL, and 14 (4.8%) isolates produced ESBL and AmpC. No isolates showed MBL and AmpC co-production. Both isolates producing all three beta-lactamases were *K*. *pneumoniae*.

## Discussion

In our study, the prevalence of AmpC production among Enterobacteriaceae was 19.4%, which was less than the rate reported by Baral et al. (27.8%) [20]. The prevalence of drug resistance depends upon local antibiotic prescribing habits and may vary not only between different geographical locations but also between different institutes at the same location [2]. In our study, the highest numbers of AmpC producing enterobacteriaceae isolate was *K*. *pneumoniae*. However, in the study by Baral et al. no *Klebsiella* spp. produced AmpC, while *E*. *coli* was the most common AmpC producing Enterobacteriaceae (59.7%) [20]. In our study, *E*. *coli* was the second most common AmpC producing isolate with 12.07% of all *E*. *coli* isolates producing AmpC.

In our study, the rate of ESBL production was 29.6%. A higher rate of ESBL production was reported by Nepal et al. (34.5%), and a lower rate by Chander and Shrestha (13 to 17%) [2, 21]. The worldwide prevalence of ESBL production has been reported as ranging from < 1 to 74% [22]. As in our study, Kaur et al. also reported the higher number of ESBL producer to be *E*. *coli* followed by *K*. *pneumoniae*, however the rate of ESBL production was very high in comparison with our results (63.4% of *E*. *coli* and 60.3% of *K*. *pneumoniae* being ESBL positive in India) [23].

The rate of MBL production was 8.5% in our study, which was higher than the rate reported by a recent study by Nepal et al. in Nepal (4%) but lower than a study by Bora et al. (19 to 21%) [2, 24]. In contrast to our study, Nepal et al. did not report any MBL cases among outpatients [2]. In our study although the rate of MBL production was higher among inpatients—3.7% of organisms isolated from outpatients were MBL positive. Similarly, significant numbers of organisms isolated from outpatients were ESBL and AmpC positive. Detection of these superbugs in outpatients indicates widespread dissemination of these bacteria in the community. This situation is worrisome and the actual gravity of the problem can be identified by detecting drug-resistant bacteria among healthy individuals. This can be done in Enterobacteriaceae by isolation, and detection of drug-resistant bacteria from stool. The most common MBL positive Enterobacteriaceae isolated in our study were *K*. *pneumoniae* followed by *E*. *coli*, which was concurrent with the results reported by Nepal et al [2].

Rates of AmpC, ESBL, and MBL production were higher among inpatients than outpatients. A hospital environment is a place where different antibiotics are regularly used [25]. Additionally the hospital environment is heavily contaminated by drug resistant bacteria escaped from patients, acting as a source for hospital-acquired infections [25]. Health care workers are also an important source of infection, who are not only responsible to transfer drug-resistant bacteria between patients but they may also act as reservoirs [25].

We reported beta-lactamases co-production among *E*. *coli* and *K*. *pneumoniae* isolates. Multiple of these bacterial isolates co-produced ESBL and MBL or ESBL and AmpC but no Enterobacteriaceae produced both MBL and AmpC. All three beta-lactamases were detected in two *K*. *pneumoniae* isolates. Beta-lactamase co-production has also been reported by Mirja et al., however their results differ from ours on the basis that they also reported both MBL and AmpC co-production, while they did not report any all-three beta-lactamases producers [26].

In Nepal haphazard use of antibiotics is very common both in health care setting and community [27]. These might be the reason for the high level of drug resistance among bacteria in this study. *Proteus spp*. earlier classified under Enterobacteriaceae and now included in Morganellaceae have also been found to produce all three beta-lactamases [28]. This is a very worrisome situation as *Proteus* spp. are intrinsically resistant to tigecycline and if found resistant to carbapenems will be pan-resistant without any remaining treatment options [13]. In our study also *Proteus* spp. were found to produce all three beta-lactamases, with one *Proteus vulgaris* isolate producing MBL. This MBL producing *Proteus vulgaris* isolate was pan-resistant but since following up on the individual patient was out of the scope of our study, we could not report on the clinical outcome of the patient from whom this isolate was isolated.

## Conclusion

In our study, there was significant AmpC, ESBL, and MBL production among Enterobacteriaceae isolates from both inpatients and outpatients. Detection of AmpC, ESBL, and MBL production among Enterobacteriaceae isolated from community-acquired infection indicates dissemination of drug resistance in the community and is a matter of great concern. Immediate attention to formulate strategies to curb the worsening condition of drug resistance, before we reach near to the post-antibiotic era, is required.

### Limitations of the study

Due to the constraint of time and budget, we could not include genotypic techniques for the detection of AmpC, ESBL, and MBL production among Enterobacteriaceae and hence could not present a deeper insight into the mechanism of the drug resistance. We also could not use modified double disc synergy test to improve ESBL detection in a setting with coexisting beta-lactamases.
